# The Crystal Structures of the Tryparedoxin-Tryparedoxin Peroxidase Couple Unveil the Structural Determinants of Leishmania Detoxification Pathway

**DOI:** 10.1371/journal.pntd.0001781

**Published:** 2012-08-21

**Authors:** Annarita Fiorillo, Gianni Colotti, Alberto Boffi, Paola Baiocco, Andrea Ilari

**Affiliations:** 1 Dipartimento di Scienze Biochimiche, University Sapienza, Rome, Italy; 2 Istituto di Biologia e Patologia Molecolari, CNR, Rome, Italy; 3 Istituto Pasteur, Fondazione Cenci Bolognetti, Dipartimento di Scienze Biochimiche, Università di Roma “Sapienza”, Rome, Italy; Institut Pasteur de Montevideo, Uruguay

## Abstract

Leishmaniasis is a neglected disease caused by *Leishmania*, an intracellular protozoan parasite which possesses a unique thiol metabolism based on trypanothione. Trypanothione is used as a source of electrons by the tryparedoxin/tryparedoxin peroxidase system (TXN/TXNPx) to reduce the hydroperoxides produced by macrophages during infection. This detoxification pathway is not only unique to the parasite but is also essential for its survival; therefore, it constitutes a most attractive drug target. Several forms of TXNPx, with very high sequence identity to one another, have been found in *Leishmania* strains, one of which has been used as a component of a potential anti-leishmanial polyprotein vaccine. The structures of cytosolic TXN and TXNPx from *L. major* (*Lm*TXN and *Lm*TXNPx) offer a unique opportunity to study peroxide reduction in *Leishmania* parasites at a molecular level, and may provide new tools for multienzyme inhibition-based drug discovery. Structural analyses bring out key structural features to elucidate *Lm*TXN and *Lm*TXNPx function. *Lm*TXN displays an unusual N-terminal α-helix which allows the formation of a stable domain-swapped dimer. In *Lm*TXNPx, crystallized in reducing condition, both the locally unfolded (LU) and fully folded (FF) conformations, typical of the oxidized and reduced protein respectively, are populated. The structural analysis presented here points to a high flexibility of the loop that includes the peroxidatic cysteine which facilitates Cys52 to form an inter-chain disulfide bond with the resolving cysteine (Cys173), thereby preventing over-oxidation which would inactivate the enzyme. Analysis of the electrostatic surface potentials of both *Lm*TXN and *Lm*TXNPx unveils the structural elements at the basis of functionally relevant interaction between the two proteins. Finally, the structural analysis of TXNPx allows us to identify the position of the epitopes that make the protein antigenic and therefore potentially suitable to be used in an anti-leishmanial polyprotein vaccine.

## Author Summary

*Leishmania spp.* are protozoa responsible for Leishmaniases, neglected diseases killing up to 60,000 people every year. Current therapies rely mainly on antimonial drugs that are inadequate due to poor drug efficacy and safety, combined with increasing drug resistance. To overcome these problems, there is an urgent need to find new and more affordable drugs. *Leishmania* reduces the hydrogen peroxide produced by macrophages during the infection by means of the tryparedoxin/tryparedoxin peroxidase couple. The two enzymes are potentially suitable drug targets since they are both necessary for parasite survival and absent in the human host. To understand the molecular basis of peroxide reduction in the *Leishmania* parasites, we have solved the X-ray crystal structures of both enzymes. Structural analyses highlight oligomerization of the two proteins and allow the regions responsible for their interaction to be identified. Moreover, based on the X-ray structures and on electronic microscopy data present in literature for the homologous proteins from *Trypanosoma brucei*, we have generated a model of interaction between tryparedoxin and tryparedoxin peroxidase from *L. major*. From the X-ray structure and from this model, we have identified the epitopes of tryparedoxin peroxidase, which is part of a potential threecomponent vaccine that is presently being studied in animal models and in human.

## Introduction

The term “Leishmaniasis” refers to a set of infectious diseases caused by protozoan parasites of the genus *Leishmania*, transmitted via the bite of phlebotomine sandflies. According to the World Health Organization [1] as many as 12 million people are believed to be currently infected, mainly in developing countries. The poor economic outlook has dampened the engagement of pharmaceutical companies, making Leishmaniasis one of the world's most neglected diseases.

The current therapies against these infections are inadequate due to poor drug efficacy and safety, combined with increasing drug resistance [2], therefore there is an urgent need of new and highly specific drugs. The trypanothione-dependent hydroperoxide metabolism, characteristic of *Leishmania* and *Trypanosoma* species, has been recognised as a promising potential target for antileishmanial drugs since it is both absent in the host and most of its components are essential to parasite survival [3]–[8]. Indeed, these parasites lack catalase, selenium-dependent peroxidases, glutathione reductase and thioredoxin reductase, and their antioxidant defence is based on a system of enzymes that depends on the unique dithiol trypanothione (N1,N8-bis(glutathionyl)spermidine, T(SH)_2_).

T(SH)_2_ is synthesized from glutathione and spermidine by trypanothione synthetase (TryS), and is kept in the reduced state by trypanothione reductase (TR) [9], [10], [11]. T(SH)_2_ participates in crucial thiol-disulfide exchange reactions and serves as electron donor in different metabolic pathways, from synthesis of DNA precursors to oxidant detoxification. The T(SH)_2_/TR system replaces many of the antioxidant and metabolic functions of the glutathione/glutathione reductase (GR) and thioredoxin/thioredoxin reductase (TrxR) systems present in other organisms and, therefore, is necessary for the parasite survival [4], [6].

One of the major biological functions of the trypanothione pathway is to regulate oxidative and, probably, nitrosative stress by shuttling reducing equivalents from NADPH to hydroperoxides and peroxynitrites [12], [13]. Two enzymes, i.e., tryparedoxin (TXN), a thiol disulfide oxidoreductase, and tryparedoxin peroxidase (TXNPx), a 2-Cys peroxiredoxin (Prx), exert a concerted trypanothione peroxidase activity analogous to that of mammalian glutathione peroxidase alone. Besides peroxides detoxification, TXN and TXNPx have a key role in DNA biosynthesis and maybe in DNA replication, mediating the activity of ROS (Reactive Oxygen Species) in metabolic regulation. In fact, TXN is likely to reduce ribonucleotide reductase, while the TXN-TXNPx pair has been reported to control the redox state of the transcription factor UMSBP (universal minicircle sequence binding protein) and consequently its binding to DNA, although recently mitochondrial redox metabolism has been proposed to be independent of mitochondrial TXN activity [14]–[17]. In fact, two homologous forms of TXN have been found, in the cytosol and in the mitochondrion (indicated as “1” and “2”, respectively), but only the cytosolic enzyme has been found to be essential [4], [17].

*Leishmania* spp. possess more than one tryparedoxin-dependent peroxidases (8 in *L. major*, 3 in *L. infantum* and *L. brasiliensis*). TXNPxs are not only good candidates to develop drugs against Leishmaniasis, but are also used to produce vaccines. In fact, three vaccine-candidate antigens (LmjF15.1140 TXNPx, LmSTI1 and LeIF) have been selected based on their abundance, immunogenicity, presence in both amastigote and promastigote forms of the parasite and conservation among most *Leishmania* species that cause human disease. The three antigens have been fused to develop a vaccine against cutaneous and mucocutaneous Leishmaniasis which is presently being studied in animal models and in human (Phase II clinical trials) [18], [19]. *Lm*TXNPx, cloned in the present study from the LmjF15.1140 gene, belongs to the Prx1/AhpC peroxiredoxins (Prx) subfamily [20], able to reduce H_2_O_2_, organic hydroperoxides and peroxynitrite thanks to redox-active cysteines. In particular, *Lm*TXNPx is a typical 2-Cys Prx and forms an obligate homodimer, whose active sites are formed by the N-proximal peroxidatic cysteine (Cp) from one subunit and a C-proximal resolving cysteine from the other (Cr') [21].

According to the generally accepted mechanism, TXN and TXNPx participate in two distinct reactions. Oxidized TXNPx first binds TXN, which reduces the intersubunit disulfide bridge (Cp–Cr'); then the reduced enzyme has to react with and process hydroperoxides.

The first reaction takes place with the formation of a disulfide bridge between the N-terminal Cys40 of TXN and TXNPx Cr' [22], with the release of Cp. This inter-protein disulfide bond subsequently undergoes nucleophilic attack by the second Cys of TXN, to leave TXNPx Cr' as a thiol or thiolate. TXN returns to the oxidized form to be recharged by T(SH)_2_.

In the second reaction, the Cp thiolate is oxidized by a peroxide to sulfenic acid (-SOH) that can react with the Cr from another monomer, forming an intermolecular disulfide bridge. Analysis of the structures of several Prx proteins reveals that the transition from the reduced to the oxidized state is associated with a conformational change involving the so-called Cp loop, including both Cp and the C-terminal arm where the Cr is located. In the reduced form, Cp is part of the first turn of an α-helix, located in a narrow solvent-accessible pocket that constitutes the active site and interacts with highly conserved residues essential for catalysis (Thr49 and Arg128, *Lm*TXNPx numbering) [23], [24]. Upon oxidation, the helix partially unwinds and Cp becomes completely exposed, suitable to be attacked by Cr'. The C-terminal arm in the reduced state is arranged in a long loop and a helix that covers the active site of the partner subunit, and upon oxidation becomes disordered allowing the formation of the disulfide bridge. This transition from a fully folded (FF) to a locally unfolded (LU) conformation, essential for catalysis, has been related to changes in the quaternary structure. Most Prx proteins in the FF conformation oligomerize as decamers or dodecamers that dissociate to dimers upon transition to LU conformation, even though other combinations have been observed. For instance, the decamer is stabilized by oxidation in TXNPx from *T. brucei* [25] whereas it is the main arrangement for the recently characterized human Prx4, regardless of the redox state [26].

In this paper we report the X-ray structures of cytosolic TXN and TXNPx from *L. major*.

Analysis of the two structures unveils structural features at the basis of hydrogen peroxide reduction at a molecular level. Moreover, analyses of the electrostatic surface potential of the two proteins reveal the structural elements allowing their interaction. Based on this finding, a complete model for the interactions between the TXNPx decamer and the TXN dimer, which is a key element to understand the mechanism of peroxide reduction, has been built.

Finally, the structural analysis of TXNPx disclose the position of the epitopes that make the protein antigenic and, therefore, potentially suitable to be used as a vaccine.

## Materials and Methods

### Cloning, expression and purification

The gene of cytosolic tryparedoxin (*Lm*TXN) was amplified by purified *Leishmania major* DNA using the following oligonucleotides: TXNNterm 5′-CGTGCACACATATGTCCGGTGTC-3′, TXNCterm 5′-CGCACAGTAAGCTTACTCGTCTC-3′, cloned between the NdeI and HindIII unique sites of the pET28b expression vector (Novagen, Madison, WI) and sequenced. The gene of *L.major* tryparedoxin-dependent peroxidase (*Lm*TXNPx, GenBank LmjF15.1140) was amplified using the following oligonucleotides: TPXNterm 5′-CCACCAGCCACATATGTCCTGCGGTAAC-3′, TPXCterm 5′- CTGACTCCTGCGAAGCTTACAGGTTTACTGC-3′, cloned between the NdeI and HindIII unique sites of the pET28b expression vector (Novagen, Madison, WI) and sequenced. The sequence of *Lm*TXN is identical to that in GenBank, while *Lm*TXNPx sequence has four differences with respect to that deposited in GenBank. TXNPx sequence has been confirmed by triple cloning and sequencing.

Wild type *Lm*TXN and *Lm*TXNPx were expressed in *Escherichia coli* BL21(DE3) cells and purified as follows: cells were grown in 1 L of Luria-Bertani medium containing 30 mg/L kanamycin at 303 K to an A_600 nm_ of 0.8.

The proteins were expressed for 3 h at 303 K, upon induction with 1 mM isopropyl-1-thio-β-D-galactopyranoside. The cells were harvested by centrifugation (4000 g for 10 min at 277 K). Cell pellets were frozen overnight, then resuspended and sonicated in 10 mL of 20 mM Tris-HCl buffer, 500 mM NaCl, 5 mM imidazole, pH 7.5, containing 1 mM phenylmethylsulfonyl fluoride (PMSF). The extracts were then centrifuged at 16,000 g for 20 min at 277 K. The resultant supernatants were then applied to a Ni-NTA column (5 ml, GE Healthcare) equilibrated in 20 mM Tris-HCl buffer, 500 mM NaCl, 5 mM imidazole pH 7.5, containing 1 mM PMSF. The column was washed with the same buffer, and the recombinant proteins were eluted with a linear gradient of imidazole from 5 mM to 0.5 M. To remove the His tag, the peaks containing *Lm*TXN or *Lm*TXNPx were subsequently dialyzed against 20 mM Tris-HCl, 150 mM NaCl, 1 mM MgCl_2_, pH 8.4, cut with thrombin for 2 hours, dialyzed vs. 5 mM Tris-HCl buffer at pH 7.5, and loaded onto a HiTrap Q (5 ml, GE Healthcare) column, equilibrated with the same buffer, at a flow rate of 1 mL/min. The purified proteins were eluted with a linear gradient of NaCl (5–500 mM), dialyzed against 20 mM Tris-HCl buffer, pH 7.5 and concentrated with Amicon YM-10 membranes.

Both *Lm*TXN and *Lm*TXNPx were highly expressed under the experimental conditions tested, and represented about 20% of total protein content of the *E. coli* pellet. The enzymes were highly soluble and the procedure yielded about 10 mg of purified enzyme per liter of culture. The purified proteins gave a single band on SDS-PAGE. The protein concentrations were determined spectrophotometrically using the theoretical molar extinction coefficients of 29500 M^−1^ cm^−1^ for *Lm*TXN and 25500 M^−1^ cm^−1^ for *Lm*TXNPx, at 280 nm and pH 7.5.

### Crystallization, data collection and processing

*Lm*TXN and *Lm*TXNPx crystals were grown by the hanging drop vapour diffusion method at 293 K. *Lm*TXN sample was concentrated to about 8 mg/mL. The crystallization drops consisted of 1.5 µL of protein solution mixed with an equal volume of the reservoir solution on a cover slip which was suspended over a reservoir containing a solution of 20–24% (w/v) PEG 3350, 100 mM Tris-HCl (pH 8.5) and 50 mM MgCl_2_. Crystals grew in 1–2 weeks but the one used for the diffraction experiment was taken 4 months after drop set up. The crystal was cryo-protected in a solution containing the mother liquor and PEG 200 (25%, w/v).

*Lm*TXNPx sample was concentrated to about 12 mg/mL. Aliquots (1.0 µL) of protein solution with 50 mM DTT were mixed with an equal volume of the reservoir solution composed of 22–26% (w/v) PEG3350, 100 mM Bis-tris propane (pH 8.0) and 0.2 M KSCN. Little, regular, hexagonal prism-shaped crystals grew in few hours at 277 and 293 K but nearly no diffraction was detected. 6 months after drop set up, a different crystal form appeared at 293 K that diffracted at 3.0 Å resolution. This crystal was used to solve the *Lm*TXNPx structure. Crystals of the same crystal form (C2221) have also grown in a few days, but have diffracted at lower resolution. The crystal was cryo-protected by adding 20% glycerol (v/v) to the mother liquor, mounted in nylon loops and flash-frozen by quick submersion into liquid N_2_ for transport to the synchrotron-radiation source. All X-ray diffraction data were collected as 1° oscillation frames at 100 K on the beam line BL-14.1 at BESSY (Berlin, Germany) using a marCCD detector. The data were processed and scaled using HKL2000 package [27]. Crystal parameters, data-collection and refinement statistics are presented in Table 1.

**Table 1:** Crystal parameters, data collection statistics and refinement statistics of *Lm*TXN and *Lm*TXNPx.

	*Lm*TXN	*Lm*TXNPx
PDB ID	3S9F	3TUE
Space Group	C222_1_	C222_1_
Unit cell parameters (Å)		
a	33.56	113.16
b	134.66	211.47
c	70.92	90.99
No. of molecules in the asymmetric unit	1	5
Wilson B factor (Å^2^)	23.9	58.8
<B> for atomic model (Å^2^)	27.9	36.8
Resolution ranges (Å^2^)	1.8–67.0 (1.80–1.85)	3.0–50.0 (3.00–3.08)
Total observations	241519	515729
Unique reflections	14386 (1047)	21162 (1420)
Completeness (%)	98.7 (97.5)	99.8 (99.9)
Redundancy	6.7 (6.5)	2.9 (2.6)
R_merge_ a	8.5 (55.7)	15.0 (51.8)
χ^2^ b	1.15 (0.81)	1.14 (0.93)
<I/σ(I)>	21.00 (3.10)	7.30 (1.84)
R_crys_ (%)	20.1 (22.4)	19.7 (29.6)
R_free_ (%)	24.2 (30.2)	23.3 (27.8)
rms angles (°)	1.36	1.02
rms bonds (Å)	0.011	0.005
Residues in core region of Ramachandran plot (%)	138 (97.2)	800 (98.5)
Residues in generously allowed region of Ramachandran plot (%)	4 (2.8)	12 (1.4)
Residues in disallawed region of Ramachandran plot (%)	0	0

Values in parentheses are for the highest-resolution shell.

a *R*_merge_ = ∑*_hkl_*∑*_i_*|*I_i_*(*hkl*)−<*I*(*hkl*)>|/∑*_hkl_*∑*_i_I_i_*(*hkl*), where *I_i_*(*hkl*) is the *i*th observation of the reflection (*hkl*) and <*I*(*hkl*)> is the mean intensity of the (*hkl*) reflection.

b χ^2^ = ∑*_ii_*(|*I_ij_*(*hkl*)−<*I_i_*(*hkl*)>|)^2^/(σ*_i_* ^2^ *N*/(*N*−1).

### Structures solution and refinement

The structures were solved by molecular replacement, performed with the program Molrep [28]. Refinement of the atomic coordinates and displacement parameters were carried out using Refmac5 [29]. Manual fitting and model building were performed using COOT [30].

*Lm*TXN structure was solved by molecular replacement using the structure of TXN from *Crithidia (C.) fasciculata* as search model [31] (PDB code 1EWX, 62% sequence identity). The rotational and translational searches, in the resolution range 10–3.5 Å, produced a clear solution corresponding to a monomer in the asymmetric unit. Structure refinement gave an Rfactor of 0.234 and an Rfree of 0.283. Then the structure was analyzed using the TLSMD web server [32] and 8 translation-liberation-screw (TLS) groups were defined to be used in TLS refinement [33] in Refmac5, lowering the Rfactor to 0.203 and the Rfree to 0.249.

The structure of *Lm*TXNPx was solved by molecular replacement using the structure of TXNPx from *C. fasciculata* [24] (PDB code 1E2Y, 73% sequence identity) as search model in the resolution range 10–3.5 Å. The rotational and translational searches, in the resolution range 10–3.5 Å, produced a clear solution corresponding to a pentamer in the asymmetric unit. During the refinement, non-crystallographic symmetry restraints were applied between all the monomers. The model has been refined to an Rfactor of 0.199 and an Rfree of 0.233.

The quality of the models was assessed using the program PROCHECK [34]. All refinement statistics are presented in Table 1. Structural figures were generated with PyMol [35].

### Surface Plasmon Resonance (SPR) measurements

The interaction of TXN with TXNPx was studied in SPR experiments performed on a BIACORE X system (Biacore AB, Uppsala, Sweden). The sensor chip (CM5, Biacore AB) was activated chemically by a 35 µL injection of a 1∶1 mixture of N-ethyl-N′-(3-(dimethylaminopropyl) carbodiimide (200 mM) and N-hydroxysuccinimide (50 mM) at a flow rate of 5 µL/min. *Lm*TXN was immobilized on the activated sensor chip via amine coupling. The reaction was carried out in 20 mM sodium acetate at pH 6.0; the remaining ester groups were blocked by injecting 1 M ethanolamine hydrochloride (35 µL). This procedure ensures immobilization of *Lm*TXN principally via the N-terminus. As a control, the sensor chip was treated as described above in the absence of *Lm*TXN. The interaction of immobilized *Lm*TXN with *Lm*TXNPx was detected through mass concentration-dependent changes in the refractive index on the sensor chip surface expressed as resonance units (RU). The increase in RU relative to baseline indicates complex formation; the plateau region represents the steady-state phase of the interaction, whereas the decrease in RU represents dissociation of the *Lm*TXNPx from immobilized *Lm*TXN after injection of buffer. A response change of 1000 RU typically corresponds to a change in the protein concentration on the sensor chip of 1 ng/mm^2^ [36].

The experiments were carried out at 298 K in degassed 10 mM HEPES at pH 7.4, 0.15 M NaCl, and 0.005% surfactant P-20 (HBS-P buffer), or in HBS-P buffer+1 mM dithiotreitol, or in HBS-P buffer+30 mM H_2_O_2_. Measurements were performed at a flow rate of 30 µL/min with an immobilization level of TXN corresponding to about 1000 RU. Values of the plateau signal at steady state (Req) were calculated from kinetic evaluation of the sensorgrams using the BIAevaluation 3.0 software. A Scatchard analysis of the dependence of Req on the concentration of TXNPx was also performed to assess the equilibrium dissociation constant.

### Electrophoresis

One-dimensional non-denaturing gel electrophoresis experiments have been carried out with Novex 4–12% Tris-Glycine Pre-Cast Gels (Invitrogen, Life Technologies, Paisley, UK), according to manufacturer's instructions. Experiments have been carried out in reducing conditions by adding 5 mM dithiotreitol to sample; oxidizing conditions have been obtained by adding 30 mM or 300 mM H_2_O_2_ to sample. One-dimensional denaturing SDS gel electrophoresis experiments have been carried out with Novex 4–12% Bis-Tris Pre-Cast Gels (Invitrogen, Life Technologies, Paisley, UK), according to manufacturer's instructions.

### Size-exclusion chromatography

Size-exclusion chromatography experiments were performed using a Superdex 75 10/300 column (GE Healthcare) mounted on a LabFlow 4000 apparatus (LabService Analytica), using HPLC pump. The size-exclusion chromatography experiments were performed using 500 µL aliquots of protein samples equilibrated against Tris-HCl buffer 20 mM, pH = 7.5. *Lm*TXN has been loaded at two different concentrations (0.2 mg/mL and 6 mg/mL), and protein absorbance has been measured at 278 nm and 305 nm, respectively. The apparent MW corresponding to the *Lm*TXN elution peaks were calculated using BSA (Bovine Serum Albumin) (MW = 66 kDa), Horseradish peroxidase (MW = 44 kDa) and Sperm Whale Mb1 (MW = 17.6 kDa) as standards.

### Bioinformatic analyses

The dimerization interface of the *Lm*TXN dimer was analyzed using the Protein Interfaces, Surfaces and Assemblies (PISA) server [37] at the European Bioinformatics Institute (http://www.ebi.ac.uk/msd-srv/prot_int/pistart.html).

Potentially immunogenic regions of *Lm*TXNPx, were predicted by using the SEPPA (Spatial Epitope Prediction of Protein Antigens) server [38] at the Life Science of Technology School Tongji University of Shanghai (http://lifecenter.sgst.cn/seppa/).

### Peroxidase activity

The reaction between *Lm*TXNPx and H_2_O_2_ has been determined by competition approach utilizing the well known reactivity of H_2_O_2_ with horseradish peroxidase (HRP) [39],[40]. H_2_O_2_-mediated HRP oxidation is a two-electron oxidation process, leading to the formation of Compound I, which can be spectroscopically followed at 398 nm. The addition of increasing concentrations of pre-reduced *Lm*TXNPx led to a lower yield in Compound I formation.

In all experiments the enzyme was dissolved in 20 mM sodium phosphate buffer at pH 7.5. Kinetic experiments were carried out at 298 K by using a thermostated rapid mixing Applied Photophysics stopped-flow spectrophotometer (Leatherhead UK). In all experiments 4 µM HRP was premixed with different amounts of TXNPx (0, 1, 2, 3, 4 µM) in one stopped-flow syringe and mixed with an equal volume of buffered solution of 0.5 µM hydrogen peroxide, prepared from a titrated 9.1 M stock solution. All time courses were followed to completion. Each experiment is the average of three time courses.

## Results and Discussion

### TXN

#### Overall structure

**Figure 1:**
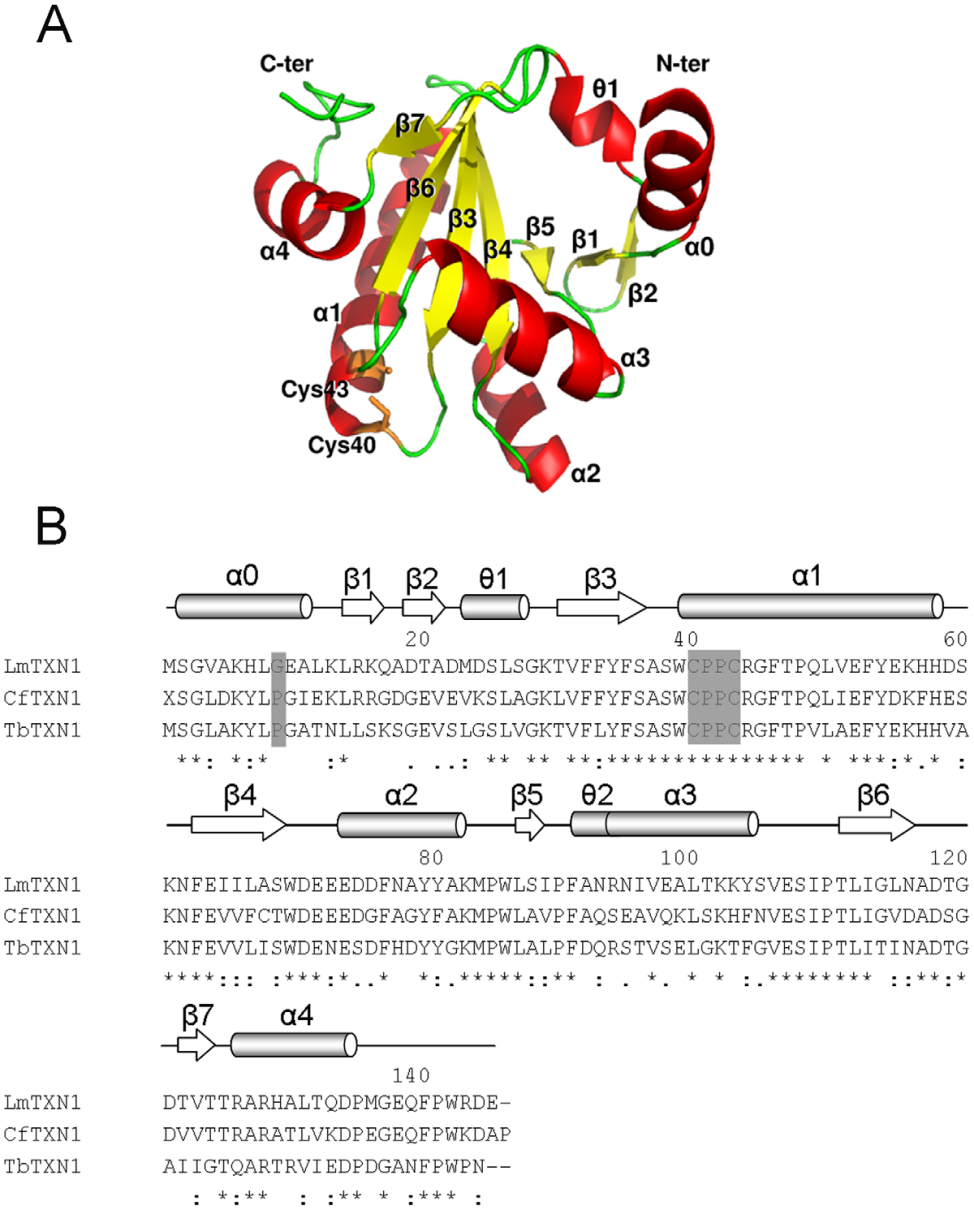
Sequence and overall structure of *Lm*TXN. **A.** X-ray structure of the monomer. **B.** Sequence alignment of *Lm*TXN, *Cf*TXN and *Tb*TXN. The alignment was performed with the program CLUSTALVIEW-MULTIALIGN (http://mobyle.pasteur.fr/cgi-bin/portal.py#forms::clustalw-multialign). The secondary structure elements are indicated (α: α-helices, θ: 3_10_-helices, β: β-strand), α0 refering only to *Lm*TXN. Asterisks indicate identical residues in the three sequences while “.” and “:” indicate “similar” and “more similar” residues as defined by the CLUSTALVIEW program. The residues of the catalytic loop are indicated. The residues in position 9 belonging to the α0 helix are also indicated in the figure.

The crystal structure of cytosolic *Lm*TXN was determined at 1.8 Å resolution. The crystal belongs to the space group C222_1_ and contains one protein molecule (residues 2–145), five Mg^2+^ ions and 66 water molecules per asymmetric unit. As shown in **Figure 1A**, the core of the structure is a seven-stranded twisted β-sheet, including parallel and antiparallel orientations, that starts with a β-hairpin β2-β1 followed by strands β5, β4 and β3 and a final β-hairpin β6-β7. The sheet is surrounded by four α-helices and two short 3_10_-helices. The active site Trp-Cys-Pro-Pro-Cys motif is located at the N-terminus of helix α1.

*Lm*TXN has a high sequence identity with TXN proteins whose three-dimensional structures are known, namely those from *C. fasciculata* (*Cf*TXN, 63%) and *T. brucei* (*Tb*TXN, 59%) (**Figure 1B** **, Figure S1**). Accordingly, all the secondary structural elements as well as the overall fold displayed by the TXN family members are conserved, apart from the N-terminus (**Figure S1**).

**Figure 2:**
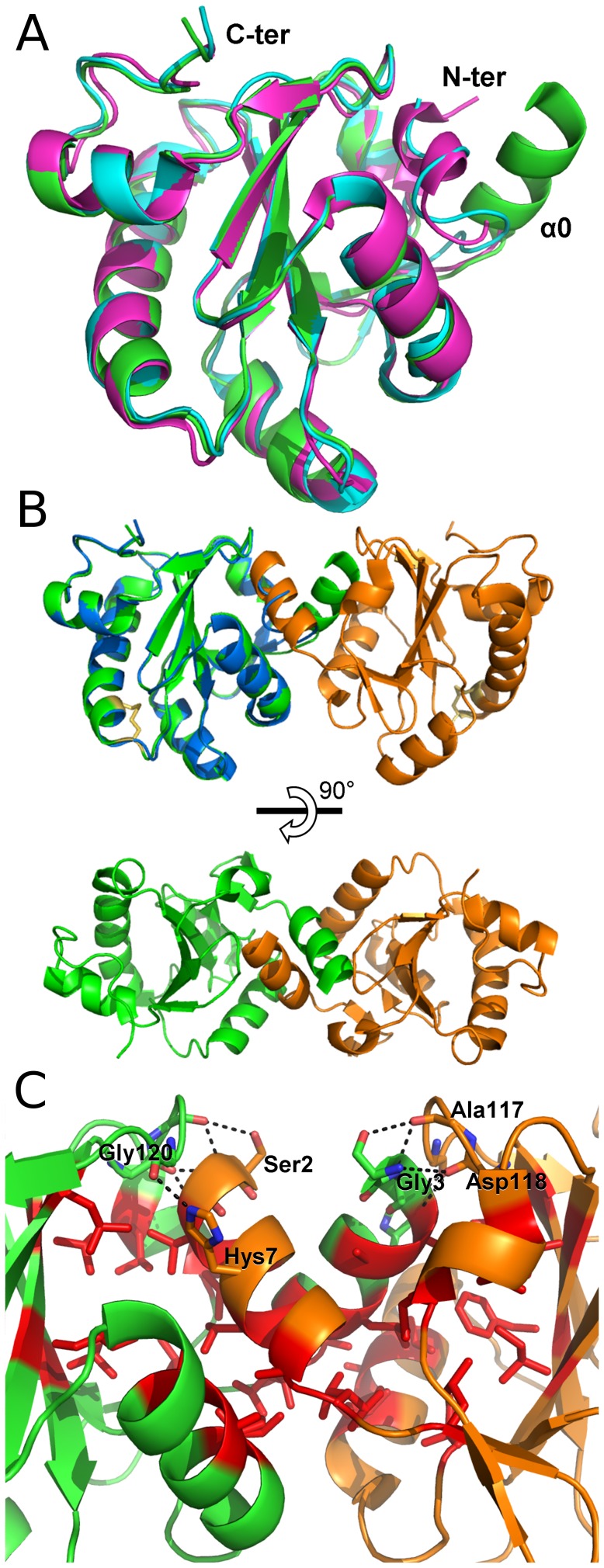
Structural comparison and dimeric assembly of *Lm*TXN. **A.** Superposition of the structures of TXN1 from trypanosomatids. Cartoon representation of *Lm*TXN (green, pdb code: 3S9F), *Cf*TXN (blue, pdb code :1QK8) [20], *Tb*TXN (magenta, pdb code: 1O73) [37]. The visible elements of secondary structure are indicated. The residues Cys40 and Cys43 of LmTXN constituting the redox active site are depicted as sticks. **B.** *Lm*TXN domain-swapped dimer. Two views of *Lm*TXN dimer, composed of two monomers that belong to distinct asymmetric units (green and orange). The superposition of *Cf*TXN (blue) to one of the *Lm*TXN monomers highlights the α0 helix-swapping. **C.** Blow up of the dimeric interface. The monomer is coloured green, the two-fold symmetry related subunit is coloured orange. The hydrophobic residues buried at the dimeric interface (Val4, Ala5, Leu8 placed on the α0-helix, Leu26, Val31, Phe33, Ile66, Ile89, Ile96, Ala99, Leu100, Leu115 and Ala117 are indicated as stick and coloured red). Residues involved in hydrogen bonds are also indicated as sticks and hydrogen bond interactions are indicated as dashed lines.

The least-square superpositions of the Cα atoms of *Lm*TXN with the corresponding Cα atoms of *Cf*TXN or *Tb*TXN gave different results whether the first residues were excluded or included in the calculation. Superpositions of the *Lm*TXN with *Cf*TXN or *Tb*TXN yielded rmsd values of 2.1 and 2.7 Å respectively, that fall to 0.7–1.0 Å when residues 2–12 were not taken into account. As shown in **Figure 2A**, in *Cf*TXN and *Tb*TXN the N-terminus is mostly in a random coil conformation with a helix-like portion, whereas in *Lm*TXN it emerges from the structure and is folded in a 11-residue long α-helix (α0). As discussed below, this helix is not completely solvent-exposed but is involved in domain swapping with an adjacent monomer in the crystal.

#### Domain-swapped dimmer

As mentioned before, *Lm*TXN was crystallized with one macromolecule per asymmetric unit, but in the crystal extensive contacts exist between two adjacent monomers, indicating a possible dimeric assembly with potential biological relevance. In fact, as reported in **Figure 2B**, two adjacent monomers (crystallographic positions x,y,z and -x,y,-z-1/2), named A and B, form a two-fold symmetry related domain-swapped dimer by exchanging their α0 helices. The interaction involves, besides α0, helices α3 and α1 and some residues from strands β4 and β3. In addition, four Mg^2+^ ions (two per monomer) are located at the interface and stabilize the dimer. The superposition of the structure of *Cf*TXN and the subunit A of *Lm*TXN shows that the swapped secondary structure element α0 of subunit B partially overlaps to the helix-like N-terminus of *Cf*TXN. To our knowledge, neither oligomerization or domain swapping have been described for TXNs. In order to discriminate between a significant interaction and an artefact from crystal packing, the dimerization interface was analyzed with the Protein Interfaces, Surfaces and Assemblies (PISA) Service [37] at the European Bioinformatics Institute (http://www.ebi.ac.uk/msd-srv/prot_int/pistart.html) and the dimer was predicted to be biologically relevant. In fact, the buried interface is 1165 Å^2^ per monomer, about 14% of the total accessible surface area (≈8000 Å^2^), is predominantly hydrophobic in nature and forms ten hydrogen bonds (**Table S1**). The hydrophobic residues buried at the dimeric interface are: Val4, Ala5, Leu8, within the α0-helix; and Leu26, Val31, Phe33, Ile66, Ile89, Ile96, Ala99, Leu100, Leu115 and Ala117. The hydrogen bonds stabilizing the interface are reported in Table S1 and involve, as shown in **Figure 2C**, residues Ser2, Gly3, Val4 and His7, placed on the α0-helix of one subunit, and residues Ala117, Asp118 and Gly120, placed on the β6-β7 loop of the two-fold symmetry related subunit. The free energy of assembly dissociation (ΔGdiss) has been estimated by PISA to be 28.5 kcal/M.

All these data point to a thermodynamically stable dimer with a highly specific binding surface. Since the effects of ligand binding on energy calculations in PISA may be quite significant [37] and lead to incorrect assignment, the analysis of the interface has been repeated by excluding Mg^2+^ ions. In this case the calculated ΔGdiss is 14 kcal/M, lower than that calculated in presence of Mg^2+^ but still indicative of a stable dimer.

Mg^2+^148 and Mg^2+^149 are placed at the interface between the two-fold symmetry related monomers stabilizing the dimers with a number of electrostatic interactions involving several water molecules, the oxygen atoms of Asn93 (O-Mg^2+^ = 3.3 Å) and His7 (Mg^2+^-O = 2.7 Å).

Native polyacrylamide gels, run in both reducing and oxidizing conditions, show that at concentrations similar to those present in the parasite, i.e. about 40 µM [5], a small fraction of *Lm*TXN exists as a dimer, independent of redox conditions (see paragraph “Oligomeric state in solution”).

In the light of the unique structural features of *Lm*TXN, attention has been focused on the sequence of the N-terminal region, corresponding to helix α0. This sequence has been compared to all the available TXN1 and TXN2 homologues (data not shown), namely those from *Leishmania mexicana* (*Lmex*), *L. braziliensis* (*Lb*), *L. infantum* (*Li*), *Crithidia fasciculata* (*Cf*), *Trypanosoma brucei* (*Tb*) and *T. cruzi* (*Tc*). An interesting difference occurs at position 9 in the sequence, located between the second and third turns of the helix (**Figure 1B**): most TXNs have proline in this position, while only *Lm*TXN1, *Li*TXN1 and *Lmex*TXN1 have glycine. Proline residues within α-helices can disrupt or alter helix conformations, as observed for example for Pro48 in the α1 helix in the known structures of TXN [22], [41]. Thus, the N-termini of TXNs having a Pro in position 9 are not likely to assume the conformation observed in *Lm*TXN. Formation of the N-terminal α-helix in *Lm*TXN may also be facilitated by the interactions between the two-fold symmetry related monomers in the dimer.

#### The active site

**Figure 3:**
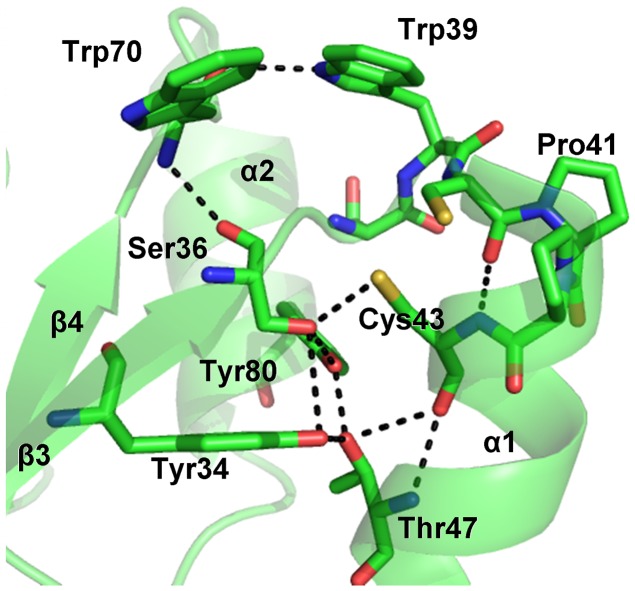
*Lm*TXN active site. The residues surrounding the two catalytic cysteine residues and the hydrogen bonds network between the residues of the catalytic site are indicated.

The environment of the active site of LmTXN is constructed by the C-terminal residues of β3, the turn that links β3 to the N terminus of α1, and residues of the loop following the C terminus of α1 and the C-terminal section of β4 (**Figure 3**).

The active site motif Trp-Cys-Pro-Pro-Cys shows the same conformation and interactions described for other TXNs [22], [41]. In particular, Cys40 is solvent exposed while the buried Cys43 participates in a hydrogen bond network comprising the highly conserved residues Tyr34, Ser36, Trp39, Thr47 and Tyr80. The side chain of Trp39, belonging to the active site motif, is held in place by a hydrogen bond with the carbonyl group of Trp70, over the Cys40-Cys43 couple and acts as a lid that covers the redox active disulfide (**Figure 3**). It should be mentioned that in the oxidized *Tb*TXN Trp39 adopts a different conformation where it is rotated toward the solvent (**Figure S2**), which is probably induced by the interaction with a symmetry-related molecule [41].

**Figure 4:**
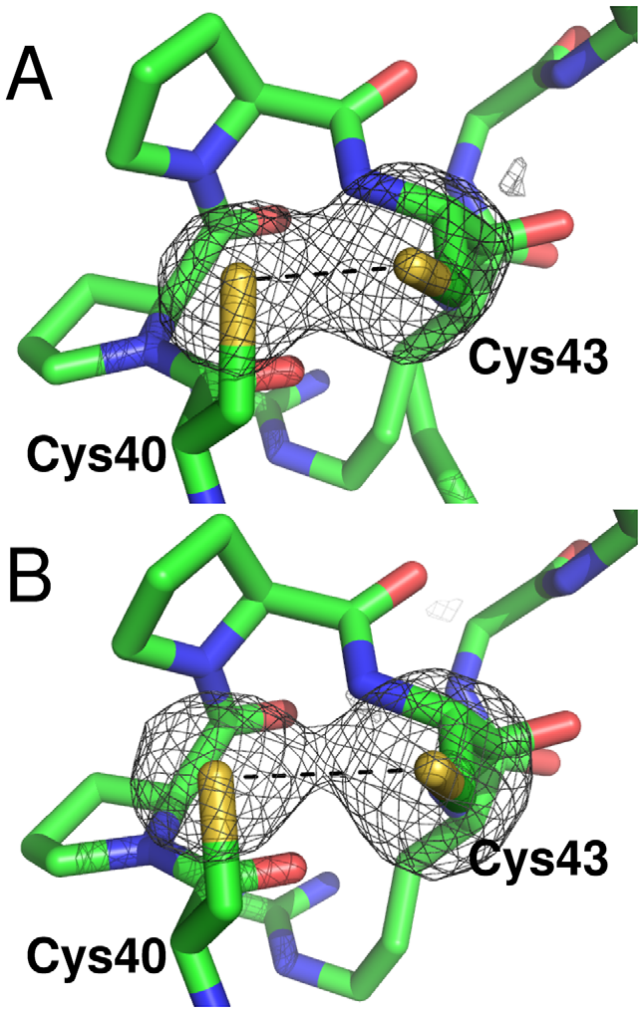
Disulfide bridge photoreduction during diffraction data collection. The omit maps Fo-Fc for the sulfur atoms in the active site of *Lm*TXN are shown. The maps have been calculated from the first 60 (**A**) and the last 150 (**B**) of 250 total diffraction images. The electron density is contoured at 4σ level. The active site CPPC motif is represented as sticks.

Interestingly, the refined S-S distance is 2.66 Å, intermediate between an oxidized (2.0–2.1 Å) and a reduced disulfide bridge (higher than 3.0 Å). Due to the high redundancy of the whole dataset collected, it was possible to split it in two subsets (the first 60 and the last 150 of 250 total diffraction images), both complete (89.2 and 91.8% respectively) and calculate two new electron density maps (**Figure 4A, 4B**). The two maps revealed S-S distances of 2.41 and 3.02 Å respectively, indicating that *Lm*TXN crystallized in the oxidized form and has been photoreduced by synchrotron radiation. Disulfide breakage takes place without any relevant structural change either in the overall structure or in the active site, that appears almost insensitive to the change in redox state. A similar behaviour had been observed in *Cf*TXN2 [**41**].

#### Oligomeric state in solution

**Figure 5:**
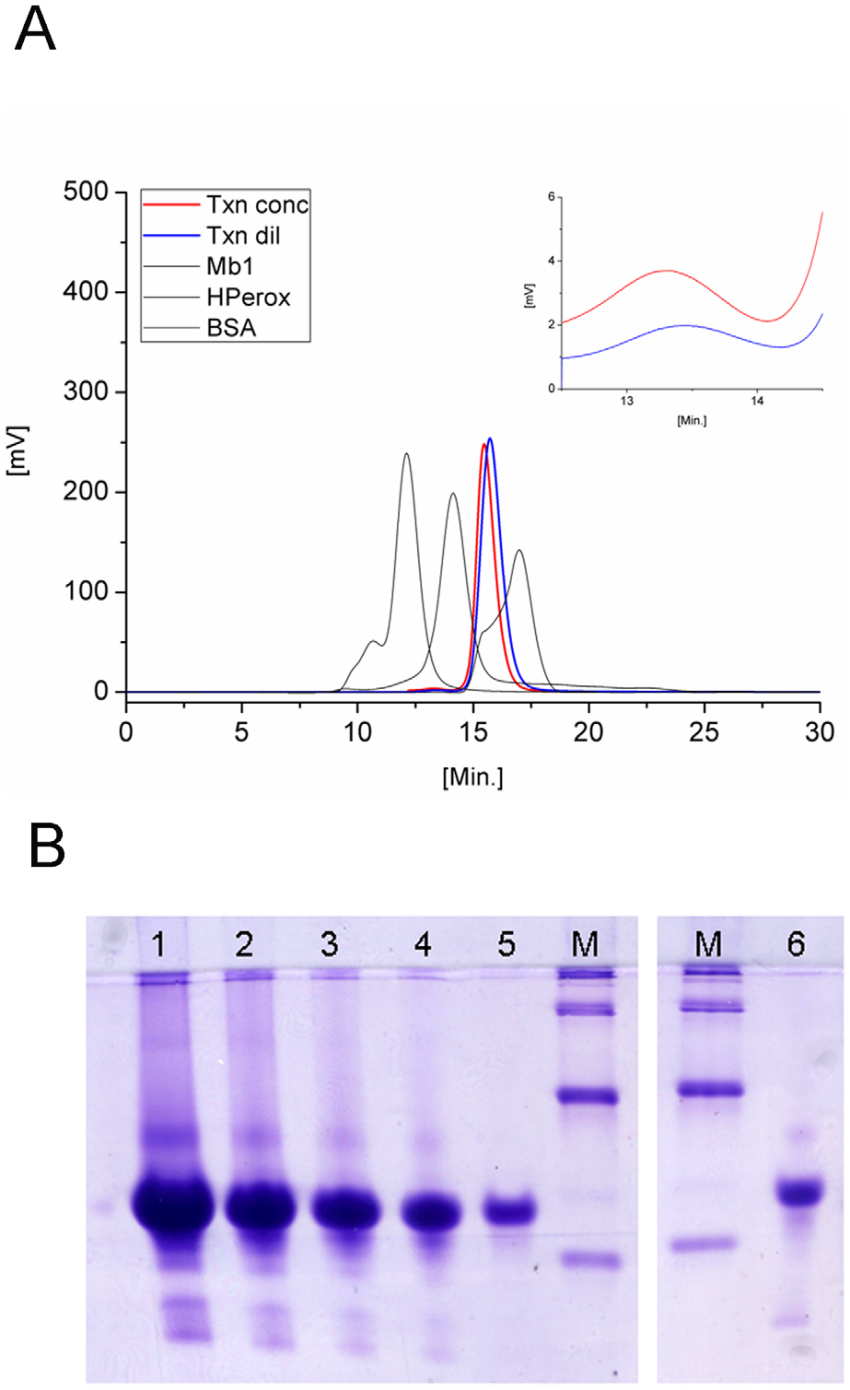
Size-exclusion chromatography and native gel electrophoresis experiments. **A.** Size exclusion chromatography experiments. The elution patterns of the standards are reported in black: peaks from left to right: Bovine Serum Albumin (BSA), Horseradish peroxidise (HPerox), and Sperm whale myoglobin (Mb1). The elution profile of diluted *Lm*TXN sample (loading concentration: 0,2 mg/mL, blue) and concentrated *Lm*TXN sample (loading concentration: 6 mg/mL, red) are also reported. The insert shows a blow up of the peak corresponding to the dimer in solution. **B.** Native PAGE of *Lm*TXN. Lanes 1–5: decreasing concentrations of TXN in Tris-HCl 20 mM pH 7.5 (8, 4, 2, 1, 0.2 mg/mL, respectively). Lane 6: TXN high molecular weight peak from size exclusion chromatography. M: NativeMark Invitrogen. Bands corresponding to about 20, 66 and 146 kDa are visible.

The oligomeric state of the *Lm*TXN was analyzed by means of size-exclusion chromatography experiments and native polyacrylamide gels. Two *Lm*TXN protein samples at low (0.2 mg/mL) and high (6 mg/mL) concentration, respectively, have been analyzed by size exclusion chromatography. The elution profile of the diluted protein (0.2 mg/mL) shows the presence of two peaks: a small peak eluting after 13.23 min and corresponding to an apparent MW of 49.3 kDa and an intense peak eluting after 15.7 min and corresponding to an apparent MW of 25.8 kDa (the MW of recombinant *Lm*TXN is 15543.5 Da whereas the MW of the protein, including the His-tag is 18706.9 Da). The increase in protein concentration of the loaded sample (6 mg/mL) causes a small shift of both elution peaks and an increment in the ratio between the two peaks (low intensity peak vs. high intensity peak) (**Figure 5A**). The high intensity peak has been assigned to the monomer whereas the low intensity peak has been assigned to a dimer. The identity of the low intensity peak has been controlled both with native (**Figure 5B** **, lane6**) and SDS denaturant polyacrylamide gels (data not shown). The native polyacrylamide gels have been run at protein concentrations between 0.2 and 8 mg/mL (8 mg/mL, 4 mg/mL, 2 mg/mL, 1 mg/mL, 0.2 mg/mL, **Figure 5B**). At high protein concentration, two bands are present; one band very intense corresponds to an apparent MW of 30–35 kDa and another, less intense, corresponds to an apparent MW of 60–70 kDa. The less intense band decreases its intensity when the protein concentration decreases and disappears when the protein sample used to run the gel is 0.2 mg/mL.

Both size-exclusion chromatography and native polyacrylamide electrophoresis experiments show that *Lm*TXN undergoes a monomer-dimer equilibrium in both oxidizing and reducing conditions (**Figure 5**), with a strong prevalence of the monomeric fraction. The dimer/monomer ratio increases as a function of the *Lm*TXN concentration; densitometric analysis of the experiment in **Figure 5** shows that at concentrations similar to the physiological ones in the *L. major* amastigote stage (about 40 µM), the protein in dimeric state is about 3% of total *Lm*TXN, and increases further (up to 6% in lanes 1 and 2) at higher concentrations. This observation suggests that *in vivo*, under conditions of high *Lm*TXN expression, which occur when the parasite lives inside the macrophage as amastigote, the protein can exist as a dimer.

### TXNPx

#### Overall structure

**Figure 6:**
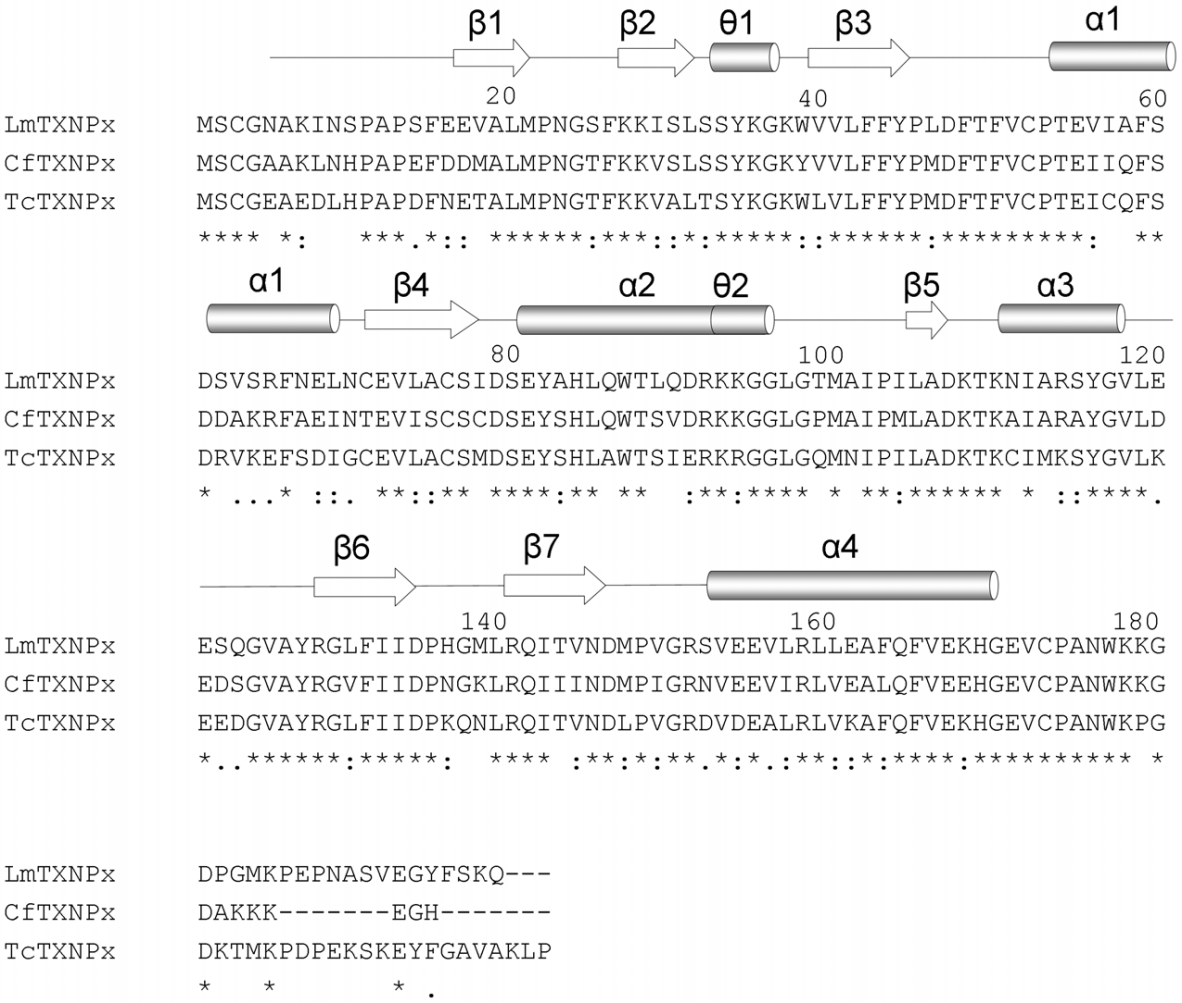
Sequence alignment of *Lm*TXNPx, *Cf*TXNPx, TcTXNPx. The alignment was performed with the program CLUSTALVIEW-MULTIALIGN (http://mobyle.pasteur.fr/cgi-bin/portal.py?form=clustalw multialign). The secondary structure elements are indicated (α: α-helices, θ: 3_10_-helices, β: β-strand), α0 referring only to *Lm*TXN. Asterisks indicate identical residues in the three sequences while “.” and “:” indicate “similar” and “more similar” residues, respectively, as defined by the CLUSTALVIEW program.

The structure of *Lm*TXNPx was determined at 3.0 Å resolution. The crystal belongs to the space group C222_1_ and the asymmetric unit contains five protein molecules (A, B, C, D, E), modelled from residue 5 to 168 (4–169 in A), and 64 water molecules. Although the sequence similarity between TXN and TXNPx is very low, the two enzymes share a common overall fold, typical of the thioredoxin superfamily. In fact, similarly to TXN, one monomer of TXNPx consists of a seven-stranded twisted β-sheet (β2, β1, β5, β4, β3, β6, β7) surrounded by four α-helices and two short 3_10_-helices, (**Figure 6**). The peroxidatic cysteine Cys52 is placed at the N-terminus of the kinked α-helix α1, in a location resembling that of Cys43 in TXN. The so-called resolving cysteine Cys173, the second residue essential for activity, is not visible since it is located in the C-terminal portion of the polypeptide that is disordered in all the monomers.

**Figure 7:**
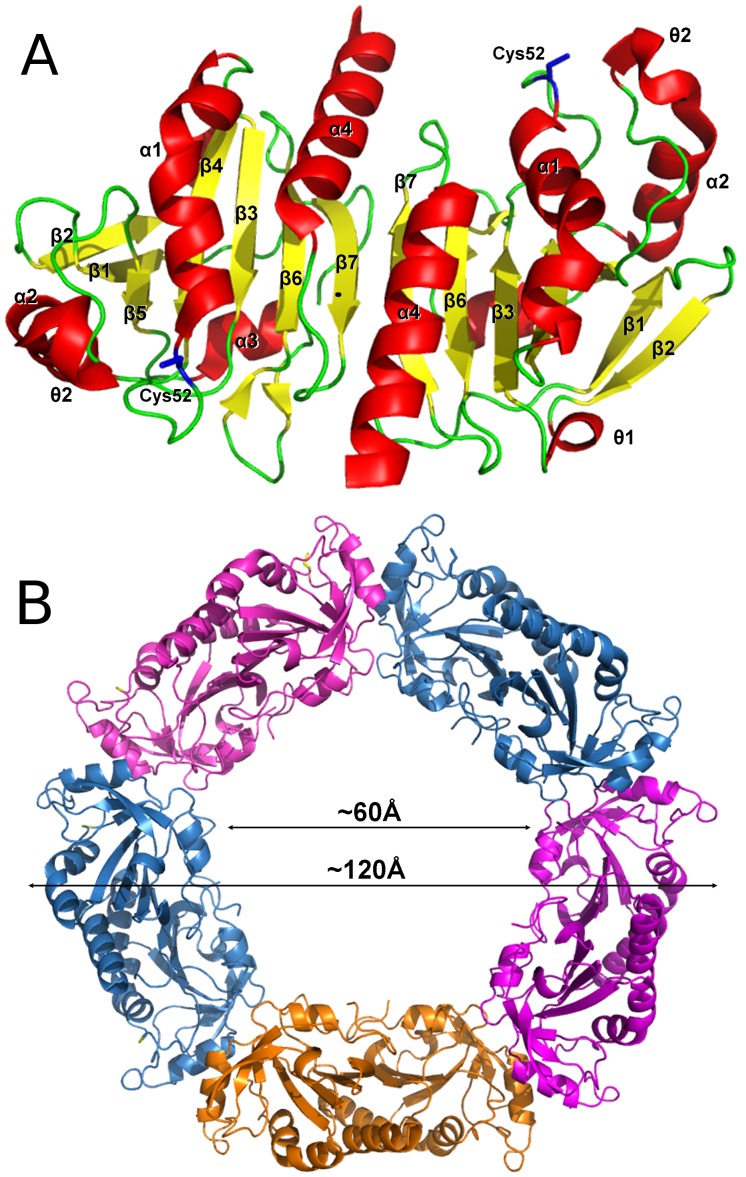
Overall structure of *Lm*TXNPx. **A.** Three-dimensional structure of *Lm*TXNPx dimer. Secondary structure elements are indicated. **B.** Decameric assembly of *Lm*TXNPx. The five dimers assembled to form the decamer are highlighted by different colours.

The *Lm*TXNPx subunits, as for *Lm*TXN, are associated in homodimers (α_2_); however, they are arranged in a different way. In TXNPx the interaction involves mainly residues from the β7-strand so that the β-sheets of two subunits are aligned to form a single 14-stranded β-sheet (**Figure 7A**). The dimers, in turn, are organized in pentamers (α_2_)_5_ and the resulting quaternary structure corresponds to a toroidal decamer formed by two adjacent asymmetric units, with outer diameter of ∼120 Å and inner diameter of ∼60 Å (**Figure 7B**). Interestingly, native polyacrylamide gels run in both reducing and oxidizing conditions show that this quaternary assembly is maintained also in solution, both in oxidizing and reducing conditions (**Figure S3**). This finding is not unprecedented since Cao et al. (2011) [24] have shown that human Prx4, belonging, like *Lm*TXNPx, to the peroxiredoxin superfamily, conserves the decameric state regardless of the redox state as well.

The same overall fold and decameric assembly of *Lm*TXN have been found in most crystal structures of typical 2-Cys Prx proteins, including the two known TXNPxs from *C. fasciculata* and *T. brucei* (pdb codes 1E2Y and 1UUL) [23], [24].

In order to predict the stability of the decameric assembly of *Lm*TXNPx with respect to other members of the Prx family, the interface between different dimers (α_2_) in the *Lm*TXNPx decamer was analyzed with PISA [37]. The calculated inter-dimer interface is 800 Å^2^, which is higher than those of AhpC (PDB code 1YEX) (670 Å^2^), that in solution may exist both in dimeric and decameric forms [42], and Prx4 (PDB code 3TJB) (730 Å^2^), that was predicted to be a decamer in solution [26]. The interaction surface between dimers at the decameric interface of *Lm*TXNPx is also more extended than those of *Cf*TXNPx (730 Å^2^) and *Tc*TXNPx (700 Å^2^), crystallized with a decameric assembly.

The main structural differences among the various Prx proteins concern two regions: the Cp loop, containing the peroxidatic cysteine, and the C-terminal arm that includes the resolving cysteine (**Figure S4**). This structural variability is related to the redox state and reflects the structural switch between FF and LU conformations that occurs during catalysis.

The most immunogenic regions of *Lm*TXNPx have been predicted by using SEPPA [38], based on crystal structure. The predictions have been done at the monomer, dimer and decamer level. The most important epitopes have been proposed to be in the monomer: GNAKINSPAPSFE 4-16, SLSSYKG 30-36, KK 93-94, RSYGVLEESQGV 114-125, DPHGM 134-138, Q 164, VEK 166-168; in the dimer: SPAPSFE 10-16, SLSSYKG 30-36, KK 93-94, R 114, LEESQG 119-124, PHG 135-137, EK 167-168; in the decamer: SPAPSFE 10-16, SLSS 30-36, KG 32-33, KK 93-94, ES 118-119, PHG 132-134, EK 164-165.

#### The Cp-loop

As already mentioned in the introduction, the transition from the reduced to the oxidized state in peroxiredoxins is accompanied by a large conformational change involving the C-terminal tail, where Cr is located, and the 43–53 loop (*Lm*TXNPx numbering) that harbours Cp (Cp loop). The protein in the reduced state is frozen in the so called FF conformation where Cp resides in the first turn of an α-helix and points towards the narrow solvent-accessible pocket of the active site, whereas the Cr-containing C-terminal arm is arranged in a long loop and one helix that covers the active site of the partner subunit. The transition from FF to LU conformation has also been related to changes in the quaternary structure, i.e. to the dissociation of the decameric assembly into 5 dimers (for a review, [43]).

**Figure 8:**
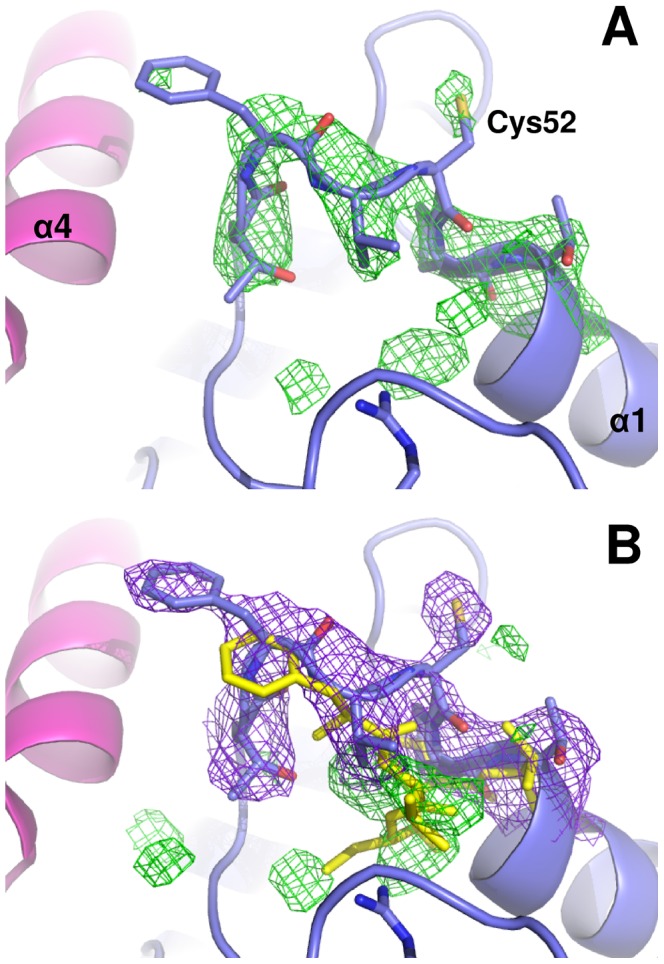
Conformation of the Cp loop. **A.** The Fo-Fc omit map contoured at 3σ (in green), calculated excluding the residues 49–54. The 49–54 residues are indicated in blue. **B.** 2Fo-Fc map calculated after building the loop 49–54. The residual electronic density (contoured at 1σ) is coloured green. The two X-ray structures of *Lm*TXNPx and *Cf*TXNPx (PDB code 1E2Y), superimposed using Coot, are coloured in blue and yellow, respectively.

*Lm*TXNPx was crystallized under reducing conditions which stabilizes the FF conformation. Interestingly, in all monomers of *Lm*TXNPx the LU conformation appears to be predominant. As shown in **Figure 8A**, the Fo-Fc omit map, calculated excluding the residues 49–54, clearly indicates that the residues 50–52 do not have an α-helical fold (typical of the FF conformation) and that the peroxidatic cysteine Cys52 is not buried inside the active site (as in the FF conformation) but protrudes towards the solvent. Moreover, once the loop is modelled according to the omit map, new density appears in the 2Fo-Fc map corresponding to the FF conformation adopted by residues 49–54 and observed for several Prxs in the reduced state (**Figure 8B**). However it has been not possible to model a satisfactory alternative FF conformation, suggesting that the LU conformation is the most populated. The LU conformation of the active site is associated with an unfolded C-terminal tail, which comprises the resolving cysteine Cys173. This residue is not visible in the structure since the electron density map allowed the model to be built up to residue 168.

In agreement with this finding, in the recently reported crystal structures of human peroxiredoxin 4 the Cp loop adopts either the LU or FF conformation independent of its redox state [44]. Moreover, in the structure of reduced *C. fasciculata* TXNPx (pdb code: 1E2Y, [24]), only in three out of the ten monomers forming the decameric assembly the Cp loop assumes the conformation expected for the reduced enzyme (FF conformation). Conversely, in three of the remaining monomers the Cp loop is in the conformation expected for the oxidized enzyme (LU conformation), and in the other four it presents an intermediate state between oxidized and reduced forms. Interestingly, Saccoccia et al. [45] reported that in the 2-Cys peroxiredoxin from *Schistosoma mansoni* the Cp loop conformation in reducing conditions may depend on pH value. Thus, at acidic pH, the Cp loop is exposed to the solvent due to the breakage of the salt bridge between Arg128 and Cys52 (*Lm*TXNPx numbering).

The B-factor analysis performed on the *Lm*TXNPx structure shows that the mean B-value of the whole structure is 36 Å^2^ whereas the mean B value of the Cp loop (residues 49–54) is 60 Å^2^ for the A monomer and 45 Å^2^ for the B monomer, indicating a high mobility of the loop.

Although growth of *Lm*TXNPx crystals takes place under reducing conditions, occurrence of a partial oxidation of the protein in the crystal cannot be ruled out. As a final consideration, the structural analysis shows that the LU conformation is favoured in the *Lm*TXNPx crystal by packing interactions. In fact, if the crystal symmetry found for *Lm*TXNPx were applied to the structures of any reduced Prxs in the FF conformation, e.g. *Tc*TXNPx, the folded C-termini from two out of the five subunit (B and E) would overlap to the C-termini of adjacent decamers.

#### *Lm*TXNPx peroxidase activity

To test the catalytic activity of the recombinant *Lm*TXNPx the horseradish peroxidase (HRP)-H_2_O_2_ competition assay was carried out, according to Trujillo and co-workers [40]. As shown in **Figure S5**, *Lm*TXNPx concentration has been increased from 0 (top trace) to 2 µM (bottom trace) after mixing, while leaving HRP concentration constant (2 µM after mixing). The time courses were fitted to a second-order equation which takes into account the fact that the reagents are not in pseudo-first order conditions [46]:
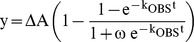
where ΔA is the observed absorbance change, k_OBS_ the observed rate constant, and ω a parameter describing the deviation of the experimental conditions from pseudo-first order. As TXNPx concentration is increased, the amplitude corresponding to compound I formation decreases hyperbolically with a “half concentration” (i.e. the concentration of TXNPx that halves the observed amplitude) of 1.18±0.07 µM, a clear indication that TXNPx competes with HRP for H_2_O_2_ (inset). The equation which describes the decrease of the observed amplitude as a function of TXNPx concentration (solid line in the inset) is:
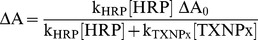
where k_HRP_ and k_TXNPx_ represent the second-order rate constant for the combination of HRP or TXNPx with H_2_O_2_, respectively, and ΔA_0_ the absorbance change in the absence of TXNPx. From this equation it is possible to determine that the ratio k_TXNPx_/k_HRP_ = 1.7. Since k_HRP_ = 2·10^7^ M^−1^ s^−1^ [47] it follows that k_TXNPx_ = 3.4·10^7^ M^−1^ s^−1^.

#### Surface Plasmon Resonance (SPR) experiments

**Figure 9:**
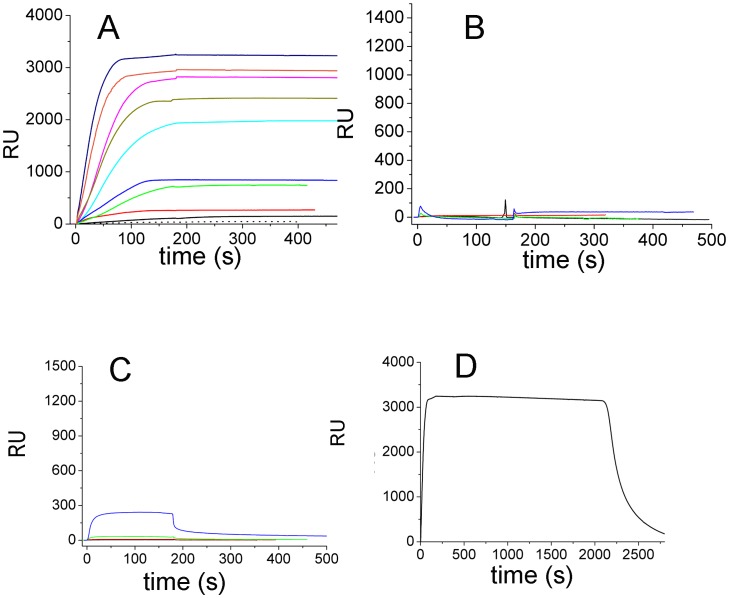
Interaction between TXN and TXNPx measured by Surface Plasmon Resonance experiments. **A.** TXNPx concentrations: 40 nM, 120 nM, 200 nM, 450 nM, 600 nM, 1.2 µM, 1.5 µM, 2.0 µM, 3.0 µM, 4.0 µM. **B.** Experiment carried out in reducing conditions (1 mM DTT). TXNPx concentrations: 40 nM, 200 nM, 1 µM, 5 µM. **C.** Experiment carried out in oxidizing conditions (30 mM H_2_O_2_). TXNPx concentrations: 80 nM, 400 nM, 2 µM, 10 µM. **D.** TXNPx 4 µM; experiment as in (A); after 2080 s, 1 mM DTT was added to the buffer.

SPR experiments show that *Lm*TXN and *Lm*TXNPx interaction takes place with a K_D_ of 1.1 µM, and that it strongly depends on redox conditions, since it is strongly decreased by the addition of either DTT or H_2_O_2_ to the buffer used for the experiment (**Figure 9A,B,C**). The dissociation rate, in particular, is very low in HBS-P buffer (5±3×10^−5^ s^−1^), while is about 9±3×10^−3^ s^−1^ in the presence of DTT (**Figure 9D**) and even higher (above 10^−1^ s^−1^) in the presence of H_2_O_2_.

SPR experiments show that the interaction between the two proteins is strongly dependent on redox conditions. This interaction is strong (K_D_ = 1.1 µM) when TXN and TXNPx are in a mixed redox state, which should mimic *in vivo* conditions, and weak when TXN and TXNPx are either fully oxidized or fully reduced, with estimated K_D_ values above 20 µM.

#### Protein interaction model

**Figure 10:**
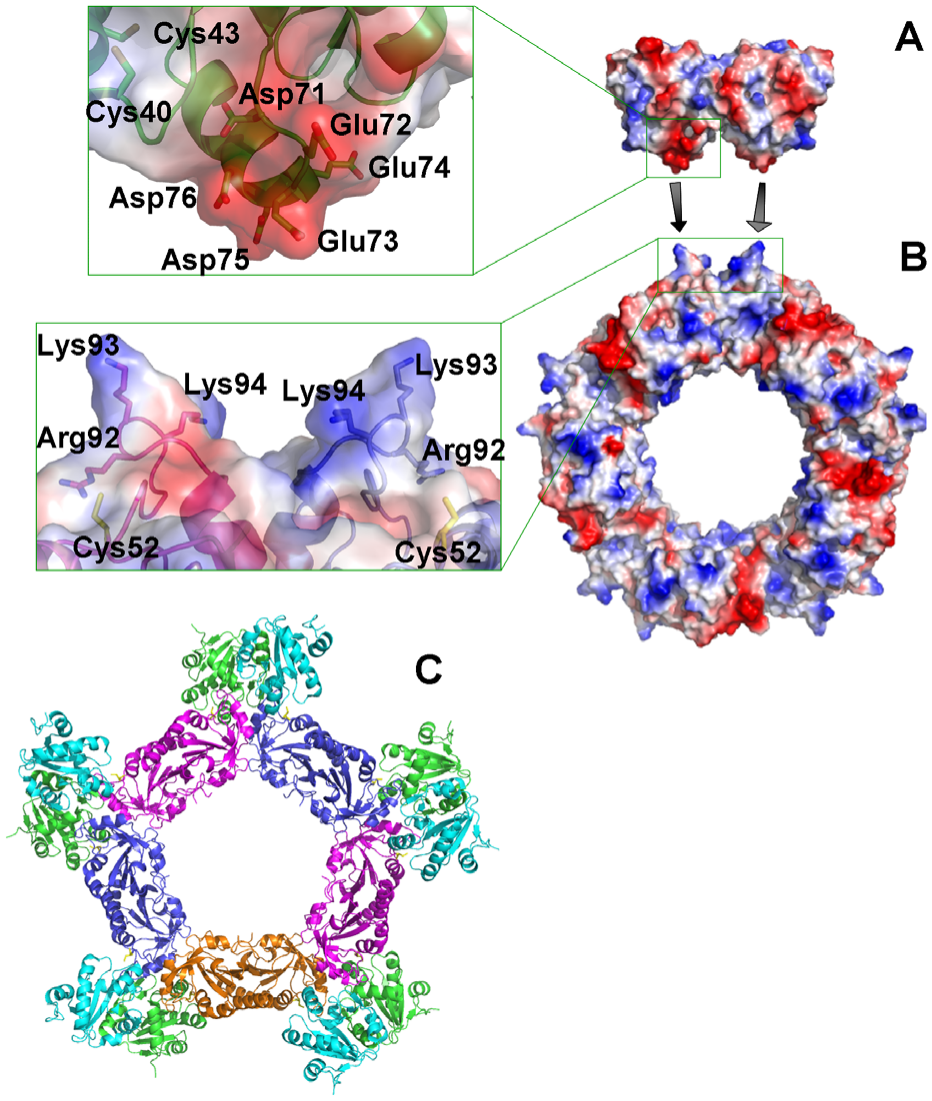
Surface charge distribution of TXN and TXNPx and model of interaction. Surface representation of (**A**) *Lm*TXN dimer and (**B**) *Lm*TXNPx decamer, coloured by electrostatic charge from red (negative) to blue (positive). The panels show the details of the regions that are supposed to mediate the interaction. (**C**) Qualitative model of interaction of the two proteins, represented as cartoons. TXN is coloured by monomer, while TXNPx is coloured by dimer, showing that each TXN dimer clasps two TXNPx dimers.

Inspection of the electrostatic surfaces of the two proteins allowed structural elements putatively involved in the interaction to be detected (**Figure 10**). The spur formed by the negatively charged residues 71–76, placed on the N-terminal part of the *Lm*TXN α2-helix and close to the two catalytic cysteines (Cys43, Cys40), may interact electrostatically with the positively charged region formed by the residues Arg92, Lys93 and Lys94 (θ2 region, see **Figure 6** and **7**) placed at the interface between two dimers of the *Lm*TXNPx decamer near Cp. As shown in **Figure 10**, a model of interaction between the two proteins based on the interaction between these oppositely charged regions fits well with the formation of a TXN dimer and is also compatible with a monomeric form of TXN, and may therefore explain how a single TXNPx decamer can be reduced efficiently by ten TXN monomers or five TXN dimers.

The model of interaction, displayed in **Figure 10C**, is in agreement with the model of Budde and Flohè based on EM data, which shows how the *Tb*TXNPx interacts with 5 TXN dimers [25]. An evidence supporting this model is that, based on both SPR experiments and data reported in the literature [25], the two proteins interact strongly when TXNPx is in an at least partially oxidized state and TXN is in the reduced state. According to our model, TXNPx binding requires TXN dissociation from trypanothione, which, in turn, takes place upon trypanothione oxidation and TXN reduction. In fact, trypanothione has been shown to interact with Glu73 [48], [31], one of the acidic residues present on the α2-helix and involved in the interaction with TXNPx as well.

The proposed model of interaction may be shared by other TXN and TXNPx homologues since the interacting regions 71–76 in TXN (*Lm*TXN numbering) and 92–94 region in TXNPx (*Lm*TXNPx numbering) are well conserved (see **Figures 1B** and **6**).

### Conclusions

The data on structural and solution properties reported in the present work reveal key structural features of *Lm*TXN and *Lm*TXNPx proteins that are relevant to their functional behavior. *Lm*TXN displays an unusual N-terminal α-helix which allows the formation of a stable domain-swapped dimer. Solution experiment indicate that a monomer-dimer equilibrium is present allowing discrete dimer formation under physiological protein concentrations. In turn, *Lm*TXNPx displays a decameric assembly both in the oxidized and in the reduced states. In particular, both the locally unfolded (LU) and fully folded (FF) conformations, typical of the oxidized and reduced protein, respectively, are populated within the reported crystal structure obtained under reducing conditions.

The high flexibility of the Cp loop with respect to the rest of the structure allows Cys52 to form easily an inter-chain disulfide bond with Cr (Cys173), thereby preventing over-oxidation which would inactivate the enzyme. This function is in agreement with the lack, in the *Leishmania* genome, of proteins homologous to sulfiredoxins, which are responsible for ATP-dependent peroxiredoxin reactivation in mammals [49], [50].

**Figure 11:**
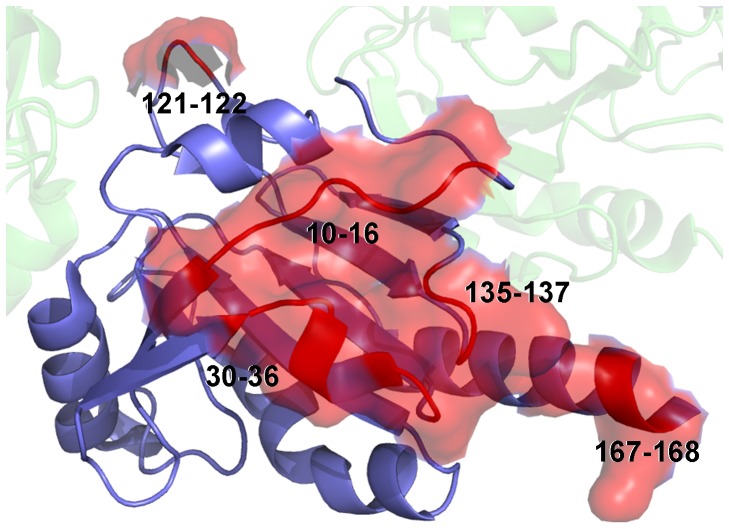
Predicted epitopes on the *Lm*TXNPx protein surface. The *Lm*TXNPx monomer is represented as ribbon. The monomer in the decamer where the epitopes are indicated is colored blue, the epitopes are colored red and indicated by residue numbers. The protein external surface, generated by PyMol, is also shown.

The present work has potential therapeutic implications, since *Lm*TXNPx has been used to develop a polyprotein vaccine against cutaneous and mucocutaneous Leishmaniasis, which is presently being studied in animal models and in humans (clinical trials, Phase II). This protein has been selected as a vaccine component on the basis of its abundance, immunogenicity, presence in both amastigote and promastigote forms of the parasite, ability to induce an immune response and conservation among most *Leishmania* species that cause human disease. The predicted epitopes of the decamer that, according to the present data and to data reported in the literature, is the predominant TXNPx assembly, are shown in **Figure 11**. Structural analysis of *Lm*TXNPx in complex with monoclonal antibodies, aimed at the identification of its antigenic determinants, is ongoing.

Some considerations should be made about the possibility to use the two enzymes as drug targets. As mentioned in the introduction section, the enzymes involved in the trypanothione metabolism are essential for parasite survival [3], [5]. In particular, the TXN-TXNPx pair, which detoxifies parasites from the ROS produced by macrophages during the infection in a T(SH)_2_-dependent manner, possess pockets surrounding the catalytic cysteines that can host small molecule inhibiting their activity. Thiophilic metals like antimony, gold and silver [10], [51], [52], have been shown to bind with high affinity to the cysteines of the *Li*TR active site and may serve as inhibitors of the TXN-TXNPx pair as well, thus resulting of potential interest as antileishmanial multi-targets agents.

## Supporting Information

Figure S1 **Ribbon diagram of TXN structure superimposed.** Native *Cf*TXN is colored in pink (PDB code 1EWX), Cys43Ala *Cf*TXN (PDB code 1O8X) in grey, *Lm*TXN in green, reduced *Cf*TXN in cyan and violet (PDB code 1O85,1O8W) and radiation damaged CfTXN (PDB code 1O7U) in yellow.
(TIF)

Figure S2 **Blow-up of the catalytic cleft in the superimposed reduced** ***Lm*** **TXN and oxidized** ***Tb*** **TXN.** The structures of the reduced LmTXN is colored green and the structure of oxidized *Tb*TXN in blue. The two cysteines and the Trp39 are depicted as ball and stick.
(TIF)

Figure S3 **Native polyacrylamide gel electrophoresis of** ***Lm*** **TXN and** ***Lm*** **TXNPx under oxidative and reducing conditions.** **A** Lanes 1, 2, 3 correspond to LmTXN = 0.3 mg/mL which has been oxidized with H_2_O_2_ = 300 mM, 30 mM and 3 mM respectively. Lines 4,5,6 correspond to *Lm*TXNPx = 0.5 mg/mL which has been oxidized with H_2_O_2_ = 300 mM, 30 mM and 3 mM respectively. **B** Lanes 1, 2, 3 correspond to *Lm*TXN = 0.3 mg/mL which has been reduced with DTT = 50 mM, 5 mM and 1 mM respectively. Lanes 4, 5, 6 correspond to *Lm*TXNPx = 0.5 mg/mL which has been reduced with DTT = 50 mM, 5 mM and 1 mM respectively.
(TIF)

Figure S4 **Variable region in the TXNPx family members.** Ribbon diagram of the LmTXNPx protein. In red are reported the variable region, in blue the conserved region. The atoms of peroxidatic cysteine are depicted as spheres.
(TIF)

Figure S5 **Competition kinetics between HRP and** ***Lm*** **TXNPx.** HRP 2 µM was exposed to H_2_O_2_ 0.25 µM in sodium phosphate buffer pH 7.5 and 298 K, either in the absence or in the presence of different concentrations of reduced *Lm*TXNPx (top to down: 0, 0.5 µM, 1.0 µM, 1.5 µM, 2.0 µM). The inset shows that as *Lm*TXNPx concentration is increased, the amplitude corresponding to compound I formation decreases hyperbolically, with a IC50 = 1.2 µM, a clear indication that *Lm*TXNPx competes with HRP for H_2_O_2_. Error bars in the inset are maximally of 0.5%.
(TIF)

Table S1 **Electrostatic interaction at the** ***Lm*** **TXN dimeric interface identified by (PISA) server at the European Bioinformatics Institute (** http://www.ebi.ac.uk/msd-srv/prot_int/pistart.html **).**
(TIF)
