# Supplementary figures and images for: The Crystal Structures of the Tryparedoxin-Tryparedoxin Peroxidase Couple Unveil the Structural Determinants of Leishmania Detoxification Pathway
Source: PLoS Negl Trop Dis. 2012 Aug 21;6(8):e1781. doi: 10.1371/journal.pntd.0001781 (PMC3424247; doi:10.1371/journal.pntd.0001781)

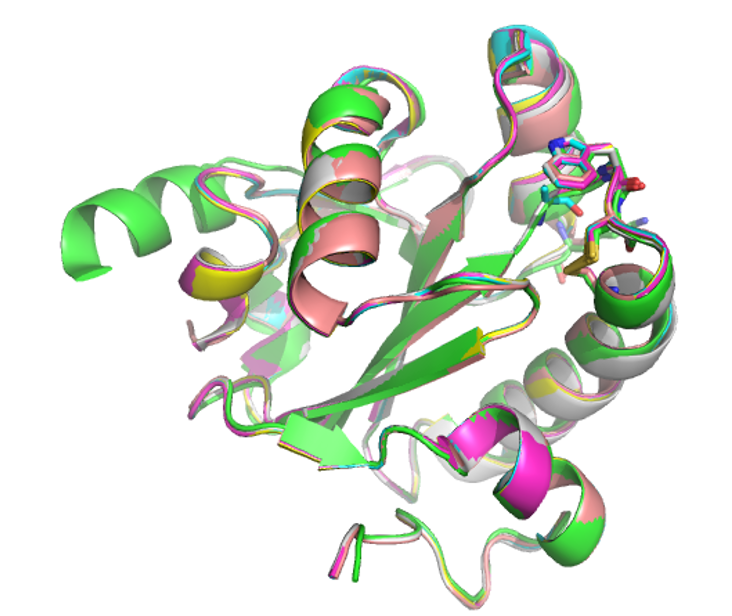

Supplement: Figure S1 — Ribbon diagram of TXN structure superimposed. Native CfTXN is colored in pink (PDB code 1EWX), Cys43Ala CfTXN (PDB code 1O8X) in grey, LmTXN in green, reduced CfTXN in cyan and violet (PDB code 1O85,1O8W) and radiation damaged CfTXN (PDB code 1O7U) in yellow. (TIF) [file pntd.0001781.s001.tif]

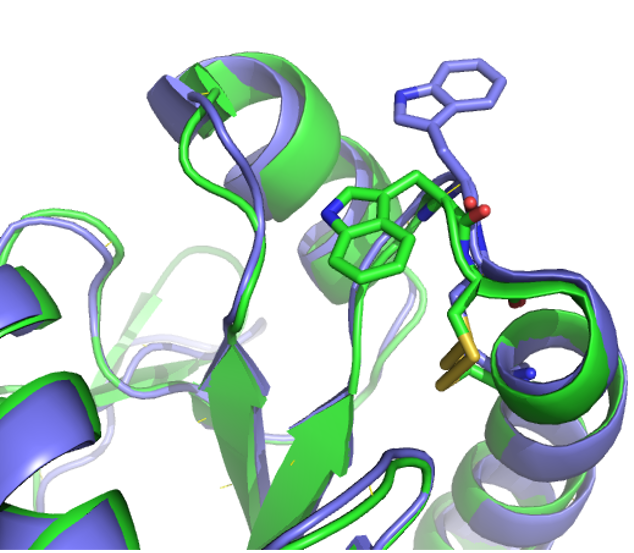

Supplement: Figure S2 — Blow-up of the catalytic cleft in the superimposed reduced Lm TXN and oxidized Tb TXN. The structures of the reduced LmTXN is colored green and the structure of oxidized TbTXN in blue. The two cysteines and the Trp39 are depicted as ball and stick. (TIF) [file pntd.0001781.s002.tif]

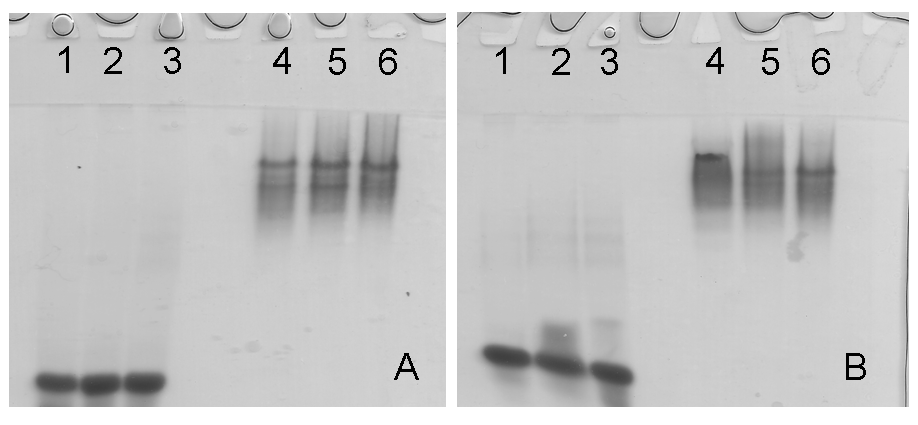

Supplement: Figure S3 — Native polyacrylamide gel electrophoresis of Lm TXN and Lm TXNPx under oxidative and reducing conditions. A Lanes 1, 2, 3 correspond to LmTXN = 0.3 mg/mL which has been oxidized with H2O2 = 300 mM, 30 mM and 3 mM respectively. Lines 4,5,6 correspond to LmTXNPx = 0.5 mg/mL which has been oxidized with H2O2 = 300 mM, 30 mM and 3 mM respectively. B Lanes 1, 2, 3 correspond to LmTXN = 0.3 mg/mL which has been reduced with DTT = 50 mM, 5 mM and 1 mM respectively. Lanes 4, 5, 6 correspond to LmTXNPx = 0.5 mg/mL which has been reduced with DTT = 50 mM, 5 mM and 1 mM respectively. (TIF) [file pntd.0001781.s003.tif]

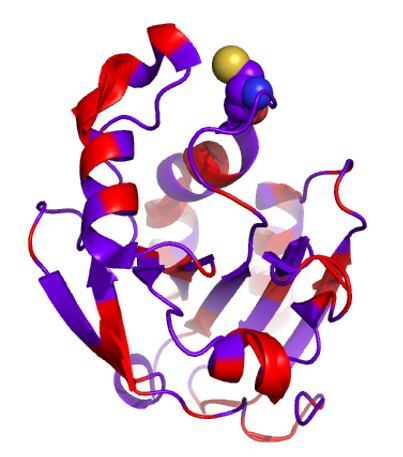

Supplement: Figure S4 — Variable region in the TXNPx family members. Ribbon diagram of the LmTXNPx protein. In red are reported the variable region, in blue the conserved region. The atoms of peroxidatic cysteine are depicted as spheres. (TIF) [file pntd.0001781.s004.tif]

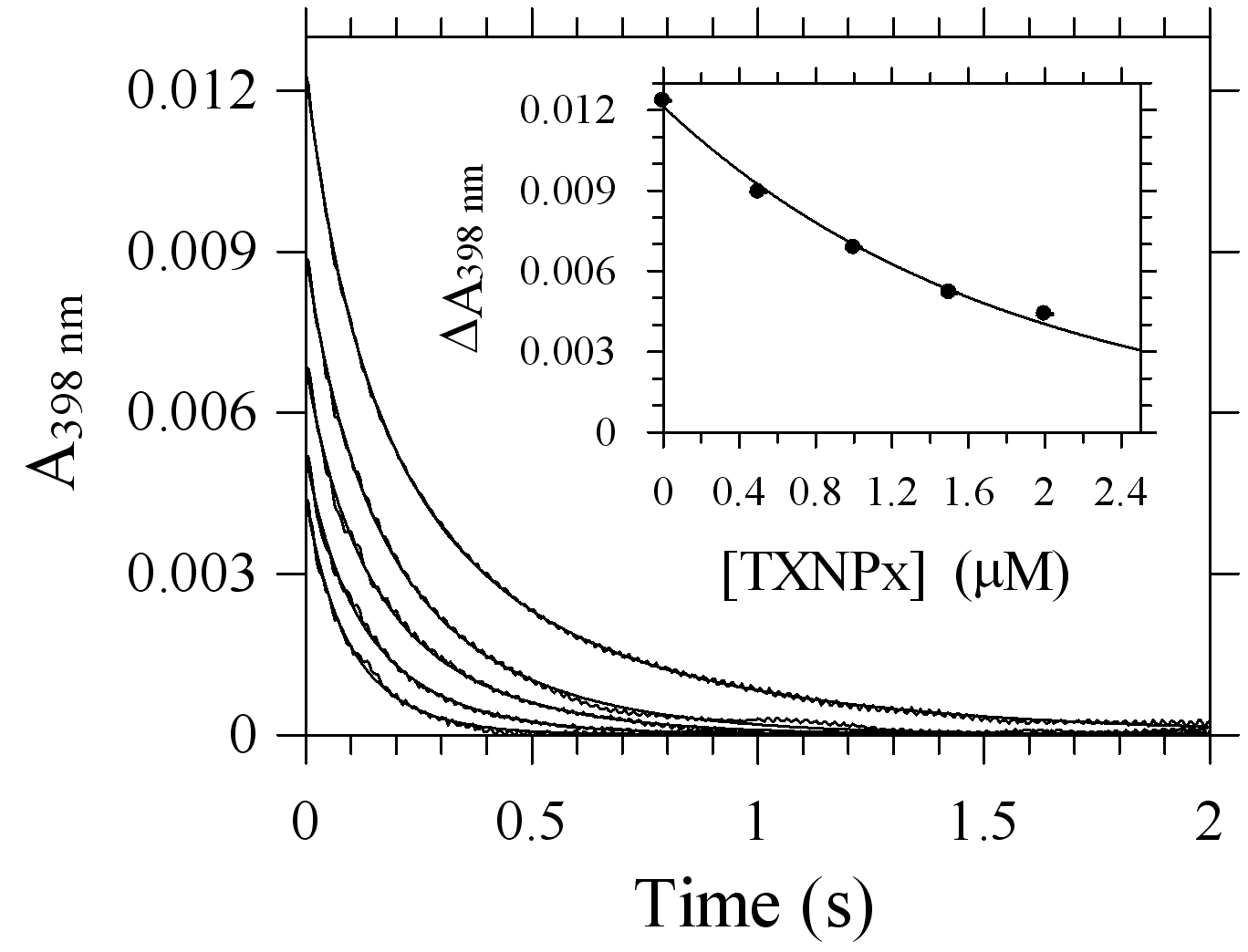

Supplement: Figure S5 — Competition kinetics between HRP and Lm TXNPx. HRP 2 µM was exposed to H2O2 0.25 µM in sodium phosphate buffer pH 7.5 and 298 K, either in the absence or in the presence of different concentrations of reduced LmTXNPx (top to down: 0, 0.5 µM, 1.0 µM, 1.5 µM, 2.0 µM). The inset shows that as LmTXNPx concentration is increased, the amplitude corresponding to compound I formation decreases hyperbolically, with a IC50 = 1.2 µM, a clear indication that LmTXNPx competes with HRP for H2O2. Error bars in the inset are maximally of 0.5%. (TIF) [file pntd.0001781.s005.tif]

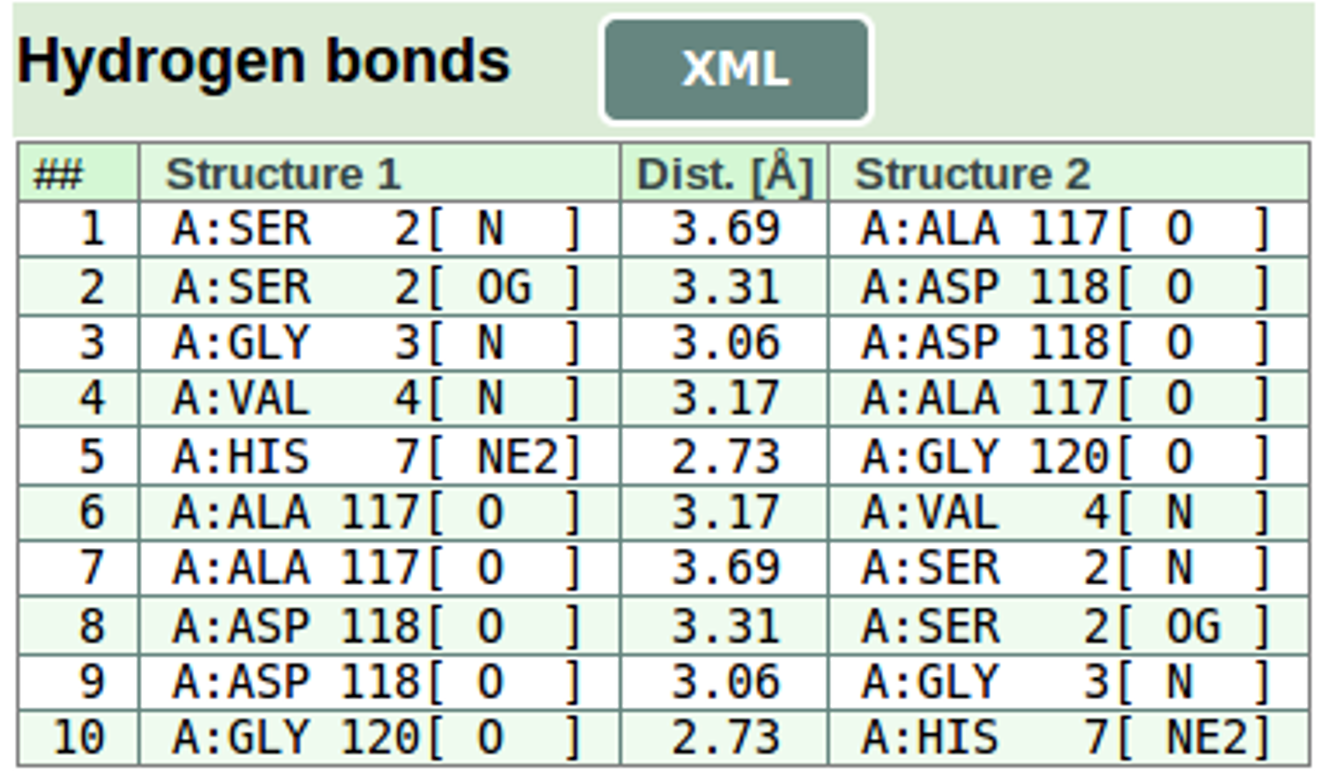

Supplement: Table S1 — Electrostatic interaction at the Lm TXN dimeric interface identified by (PISA) server at the European Bioinformatics Institute ( http://www.ebi.ac.uk/msd-srv/prot_int/pistart.html ). (TIF) [file pntd.0001781.s006.tif]
